# Microstructure and Wear Resistance of a Composite Coating Prepared by Laser Alloying with Ni-Coated Graphite on Ti-6Al-4V Alloy

**DOI:** 10.3390/ma15165512

**Published:** 2022-08-11

**Authors:** Huijun Yu, Lu Lu, Zifan Wang, Chuanzhong Chen

**Affiliations:** 1 Key Laboratory of High Efficiency and Clean Mechanical Manufacture, Ministry of Education, and National Demonstration Center for Experimental Mechanical Engineering Education, School of Mechanical Engineering, Shandong University, Ji’nan 250061, China; 2 Key Laboratory for Liquid-Solid Structural Evolution and Processing of Materials, Ministry of Education, and Shandong Engineering & Technology Research Center for Superhard Material, School of Materials Science and Engineering, Shandong University, Ji’nan 250061, China

**Keywords:** laser surface alloying, titanium alloys, nickel-coated graphite, wear resistance, solid-lubricating

## Abstract

Titanium alloys are widely used in high-tech fields, while its disadvantages such as low hardness, high coefficient of friction and poor wear resistance have restricted its applications. This study focuses on improving the friction and wear resistance of Ti-6Al-4V titanium alloys by means of laser surface alloying with Ni-coated graphite (G@Ni). The results suggest that Ni acts as a protective layer to hinder the direct contact and reaction of C and Ti in the molten pool. A part of graphite is unmelted and finally remains to form a self-lubricating wear-resistant composite coating with a compact structure. The average hardness of the coating is approximately four times that of the substrate owing to the TiC hard phase and compact microstructures as the reinforcing phase. The residual graphite in the coating plays a friction-reduction role during the wear test. The wear resistance is increased to 8.53 times that of the substrate according to wear mass loss. This study can effectively enhance the performance and expand the application of the titanium alloys by improving the wear resistance and reducing the friction.

## 1. Introduction

As one of the modern structural materials, titanium alloys are widely used in aerospace, biomedical science and the auto industry owing to the advantages of low density, high specific intensity and excellent corrosion resistance [1,2,3,4]. However, due to its low hardness, high coefficient of friction and poor wear resistance, the applications of titanium alloys are inevitably restricted [5,6,7]. Surface modification technology, such as chemical heat treatment [8], ion implantation [9], micro-arc oxidation [10], laser cladding [11], and sol-gel [12] is widely used to improve the surface properties of the substrate, thus, it has been studied to increase the hardness and wear resistance of titanium alloys, and further broaden their applications in wear occasions [13,14,15]. Among which, laser surface alloying (LSA) has attracted extensive attention from researchers because of its high processing efficiency, small heat-affected zone, controllable coating thickness, and good adhesion to substrates [16,17,18]. In the laser alloying process, under the action of a high-energy laser beam, one or more alloying elements and the surface of the substrate are rapidly melted together to form a molten pool, where a series of complicated chemical reactions take place. After rapid solidification, a coating showing an excellent metallurgical bond with the substrate forms on the surface, which is conducive to the improvement of the properties. Therefore, laser surface alloying is one of the most effective methods for the surface strengthening and modification of titanium alloys. Research on the surface strengthening and modification of titanium alloys by laser surface alloying is focused on laser carbon alloying [19], nitrogen alloying [20], boron alloying [21,22], multi-element alloying [23,24,25], etc. A previous paper showed that B_4_C/graphite could be used as a raw material to form ceramic-reinforced hard alloying coatings, increasing the micro-hardness of the substrate. The results also indicated that the Ti(C,N) phase that formed in situ in the upper coating was undeveloped dendritic, and thus reduced the possibility of crack formation in the alloying coating [26].

However, most of the current reports focus on increasing the hardness of the coating to improve its wear resistance, while research about reducing the friction coefficient, which is of vital importance as well, had been rarely studied [27]. In working conditions where it is difficult to achieve oil lubrication, self-lubricating coatings can reduce the friction coefficient, improve the wear resistance, and extend the component’s service life.

As a kind of good solid lubricant, graphite has a layered structure, with covalent bonding among the atoms in one layer with a weaker van der Waals force between different layers. There are limited reports about laser carbon alloying with graphite on the surface of a titanium alloy. The challenge is that Ti atoms have a strong affinity with C, which makes it difficult to achieve the retention of the graphite phase during the processing of the coating.

The nickel-coated graphite (G@Ni) powder used here has a spherical shell structure. Without covering with Ni, the graphite decomposes and releases C, which makes it easier to react. Compared with conventional G powder, G@Ni is expected to have a better flowability which improves the wettability with the Ti-6Al-4V substrate, and beneficial to the homogenization of the coating. Moreover, Ni is expected to act as a protective layer to hinder the direct contact and reaction of C and Ti in the molten pool. In addition, Ni would also react with Ti to produce Ni-Ti intermetallic compounds with excellent properties, such as the better toughness of NiTi and higher hardness of NiTi_2_ [27,28]. These are also expected to improve the performance of the coating. These advantages plus the self-lubricating feature will enable the application of this coating on machine components where reduced weight is required, but oil lubricating is difficult, for example, the engine valves [29]. Thus, we carried out this research to explore the feasibility of the self-lubricating graphite phase remaining in the coating prepared by laser alloying with G@Ni powder. The microstructure and wear resistance of the prepared coating are investigated.

## 2. Experimental Methods

In this study, Ti-6Al-4V was used as the substrate material. The Ti-6Al-4V titanium sheet was cut into small pieces of 10 mm × 10 mm × 20 mm and 25 mm × 25 mm × 10 mm in size, which were used for microstructure analysis, composition analysis, and micro-hardness test, as well as XRD and wear tests, respectively. The samples were abraded with SiO_2_ sandpapers. Samples were further cleaned with alcohol prior to laser alloying. G@Ni powder, formed by depositing the graphite core with a Ni shell, was used as the alloying material with the chemical composition of 75 wt.% Ni + 25 wt.% graphite and particle size ranging from 45 to 109 μm. The properties of Ti-6Al-4V and G@Ni are given in Table 1 as follows. The powder was pre-paved on the surface of the samples and the thickness of coating was controlled to 1 mm. In this experiment, a TFL-H6000 cross-flow CO_2_ laser was used for laser surface alloying, and argon was used to protect the molten pool from oxidation. The selected laser process parameters were: laser power 3500 W, laser scanning speed 300 mm/min, and spot diameter 4 mm. For the samples with the size of 10 mm × 10 mm × 20 mm, only one track of scan was performed. For the samples with the size of 25 mm × 25 mm × 10 mm in size, the entire surface was processed by scanning nine tracks with 30% of overlapping.

The 10 mm × 10 mm × 20 mm sample was cut along a cross-section in the vertical scanning direction by a wire-cutting machine, grounded through a series of sandpapers, polished with a polishing agent, and etched with a mixed solution having a volume ratio of HF:HNO_3_ = 1:3. The metallographic specimens of the coating were prepared for microstructure observation, composition analysis and micro-hardness test. The microstructure was observed by scanning electron microscope (SEM) and the elemental distribution in the coating was analyzed by the attached EMAX energy dispersive spectrometer (EDS). The micro-hardness distributions were tested by DHV-1000 Vickers micro-hardness tester with a load of 200 g and a loading time of 10 s. The samples in the size of 25 mm × 25 mm × 10 mm were grinded with a grinder. The phase composition was analyzed by MiniFlex 600 X-ray diffractometer (XRD), with a voltage of 40 kV, an electric current of 40 mA, a scanning range of 10~90°, and a scanning speed of 4°/min. The wear resistance property was tested by HT-1000 wear tester with a load of 2 kg and a rotational speed of 560 r/min. The wear counterpart was made of Si_3_N_4_. The wear mass loss was measured after the thirty-minute wear test by an electronic balance with accuracy of 0.1 mg.

## 3. Results and Discussions

### 3.1. XRD Analysis

**Figure 1:**
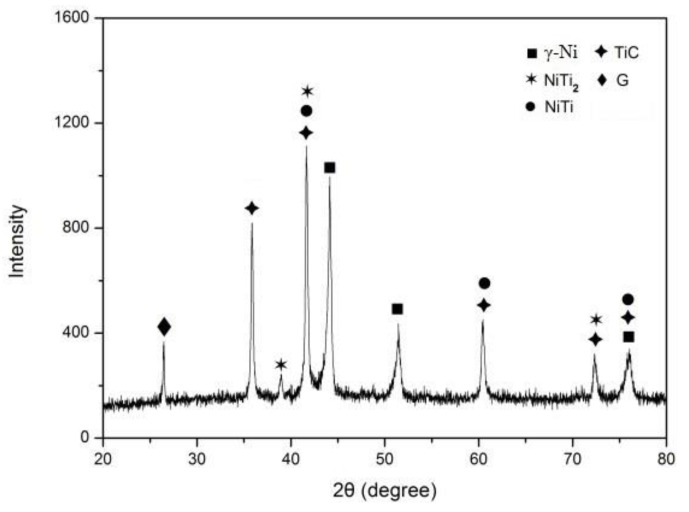
X-ray diffraction pattern of the coating.

Figure 1 shows the XRD results of the coating, indicating that the coating consists of TiC, γ-Ni, NiTi, NiTi_2_, and graphite. In the laser alloying process, complicated reactions occurred and many phases formed in situ, where Ti could react with C and Ni, forming TiC, NiTi, and NiTi_2_. Ni melted to form a molten pool, part of which reacted with Ti to form the intermetallic compounds NiTi and NiTi_2_, improving the toughness of the coating. γ-Ni had an effect of solid solution strengthening for the coating. Due to the strong affinity of Ti and C, a part of graphite reacted with Ti to form TiC, which could increase the hardness and wear resistance of the coating. The appearance of G in the coating can be attributed to the effect of Ni, which would hinder the reaction between Ti and C, resulting in graphite remaining in the coating. The presence of these phases enables the higher coating hardness and wear resistance, good plasticity and toughness, as well as a reduced friction coefficient.

### 3.2. SEM Analysis

**Figure 2:**
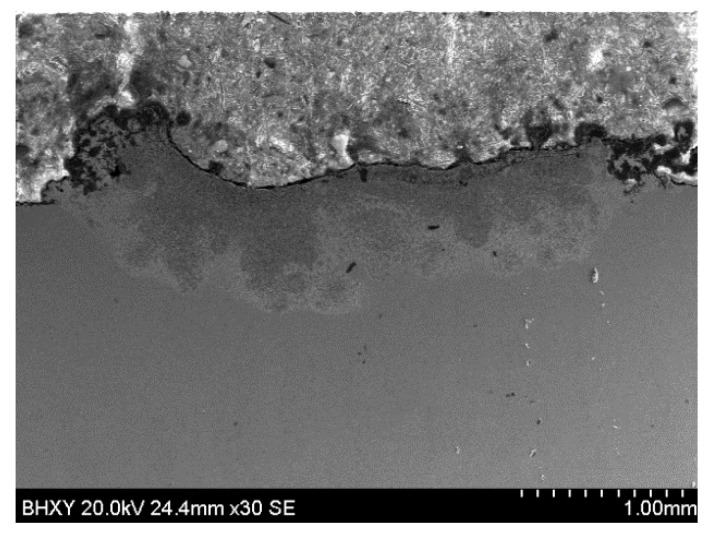
Cross-section morphology of the G@Ni single track.

Figure 2 is the cross-sectional morphology of the coating. It can be seen that there is a good metallurgical bond between the coating and the substrate without obvious cracks and pores. The coating presents a crescent shape, as a result of which the energy density of the laser spot follows normal distribution as in the following equation [30]:

$$I\left(r,0\right)=I_{0}\exp\left(-2\frac{r^{2}}{R^{2}}\right)$$

where *I* refers to the energy density near the center of the laser spot, *I*_0_ refers to the energy density at the center of laser spot, *r* refers to the distance from the center, and *R* refers to the radius of the spot. The energy density on the center of the laser spot is the highest while, at the edge of laser spot, it is lower. A proper laser energy density could fully melt the pre-paved powder to the obtained transformed coating with a fine surface quality. On the other hand, the crescent shape is also related to the convective motions in the molten pool, which consist of the constraint convective motion caused by the surface tension gradient and natural convective motion because of the horizontal temperature gradient. The melt flow which engendered these two motions follows the same direction, where the natural and constraint convective motions were coupled into a convection loop, which, respectively, rotated clockwise or anticlockwise at the right- or left-side of the molten pool. As a result, the molten pool presents a crescent shape influenced by the convection, which would be reserved owing to the chilling effect of the substrate after the laser beam moved along [31].

As there exists no surface-active elements in the molten pool, these two motions follow the same direction, where the natural and constraint convective motions were coupled into a convection loop, which, respectively, rotated clockwise or anticlockwise at the right- or left-side of the molten pool. As a result, the molten pool presents a crescent shape influenced by the convection, which would be reserved owing to the chilling effect of the substrate after the laser beam was moved away.

**Figure 3:**
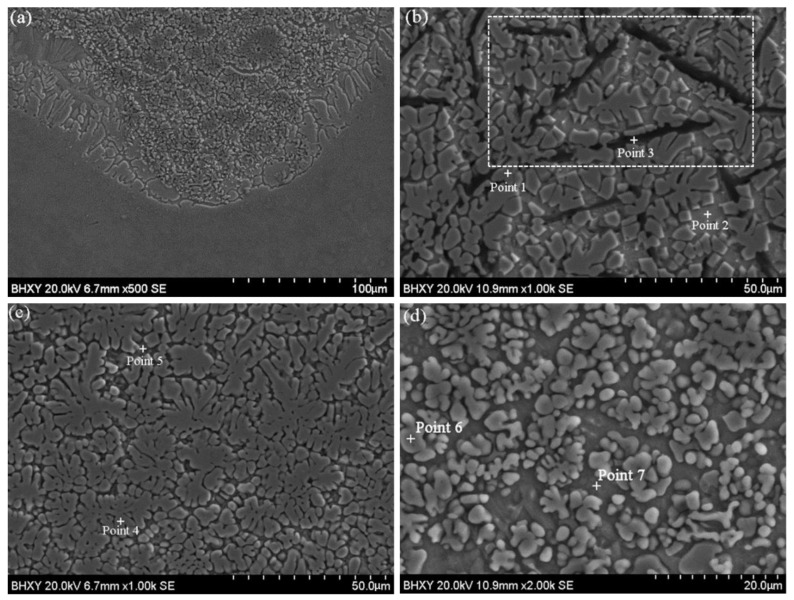
SEM morphologies of the coating: (**a**) the bonding layer; (**b**) the upper layer; (**c**) the middle layer; (**d**) the bottom layer.

The microstructures of the coating are shown in Figure 3. There is an obvious bonding line between the coating and the substrate. The coating formed a strong metallurgical bond with the substrate, where no obvious defects such as cracks and pores have been observed (Figure 3a). As is shown in Figure 3, different layers in the coating are all occupied by cellular layers, cellular-dendrites, or dendrites. The morphology is closely related to the ratio of the temperature gradient and solidification velocity. A high ratio facilitates the formation of cellular morphologies, while a low value leads to the formation of dendrites. However, the dimensions of the morphologies present a gradient distribution due to the difference of the temperature and cooling rate in the molten pool. As a result of the high laser absorptivity and low thermal conductivity of graphite, the molten pool could exist for enough time where the solidification interface moved from the bottom of the pool to the free surface. The upper layer of the coating melted first while being solidified last. Hence, molten pools close to the free surface existed for a longer time, leading to a small temperature gradient that reduces the nucleation rate. Grains at the upper parts of the coating keep growing, for which reason they appear to be coarse and non-directional dendrites (Figure 3b). At the bottom of the molten pool, due to the chilling effect of the substrate, there was not enough time for the precipitated crystals to grow (higher temperature gradient and lower solidification velocity), which efficiently weakened the feature of dendrites (Figure 3d) while enabling the growth of the directional-cellular morphologies (Figure 3a). The middle layer presents a transition of the above two, showing cellular-dendritic morphologies (Figure 3c). It is also notable that there are some black lath-like morphologies on the upper layer of the coating, which were identified to be, in Figure 3b, as discussed below.

**Figure 4:**
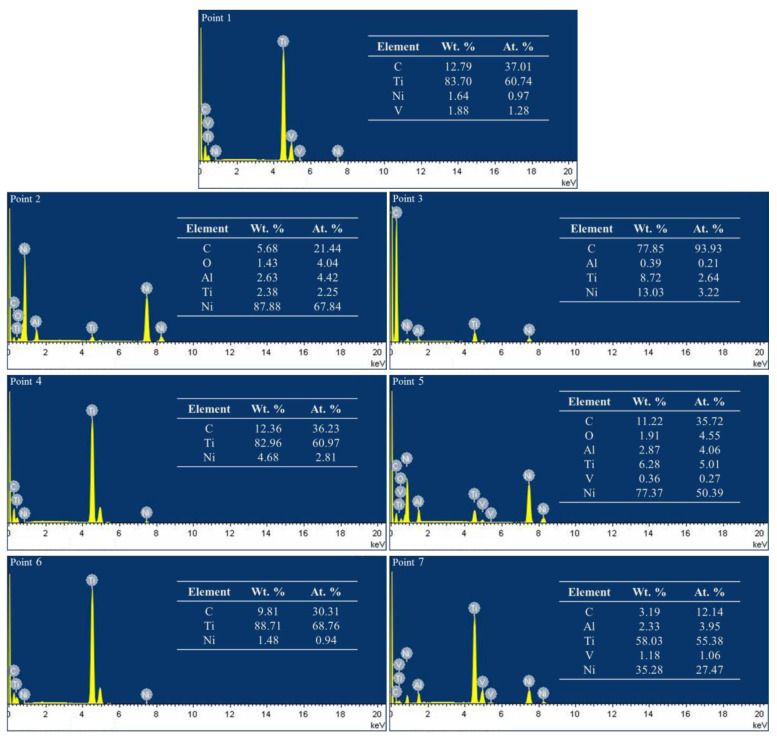
EDS results of the points in Figure 3. Leaner y axis with the full scale of 8236 cts for all the EDS spectra.

**Figure 5:**
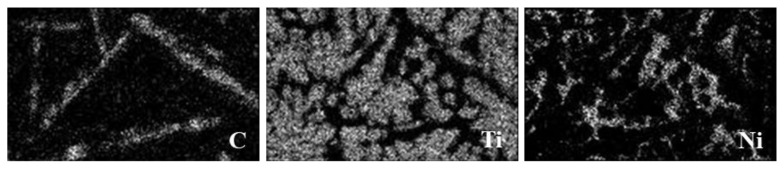
Elemental mapping of the upper layer of the coating for the selected area shown in Figure 3b.

By analyzing the composition of typical structures in the coating, the typical microstructure of the sample and the corresponding point component analysis results are shown in Figure 4. The main components of the petal-like and granular microstructure are C and Ti (Point 1, 4, 6), which can be assigned to TiC based on the XRD results. The main component of the lath-like structures is C (Point 3), which is considered as graphite based on the XRD results. The existence of graphite in the lath-like structures can be further supported by Figure 5, which shows the element mapping of the upper layer of the coating for the selected area shown in Figure 3b. It is worth noting that these lath-like structures appeared only on the upper layer of the coating. This is due to the low density of graphite, which floated up in the molten pool and aggregated on the top of the coating. In addition, the content of Ni in the matrix decreased as the depth of the coating increased (Point 2, 5, 7). It can be inferred that the Ni on the outer layer of G@Ni melted at first because of its lower melting point. In the upper and middle matrix of the coating, there was a small amount of Ti since Ti mostly reacted with C. It is thus speculated that γ-Ni is its main phase. The bottom matrix (Point 7) of the coating gathers a large amount of Ti as it is located close to the substrate. Therefore, this area is mainly composed of γ-Ni solid solutions with a relatively high content of Ti as well as NiTi and NiTi_2_, as suggested by the XRD analysis. As TiC is a cubic crystal which is similar to NaCl, the lattice planes, having low atomic arrangement densities, are more likely to form rough interfaces and grow up continuously for its low atomic coordination number to effectively expedite the growth speed [32]. The preferential and second preferential growth direction of TiC are <100> and <110>, respectively. Ni could selectively absorb on the {100} crystal plane when mixed into the molten pool quantificationally. This reduces the interfacial energy and slows down the growth rate of the {110} crystal plane, causing the petal-like TiC grains grows along the <100> direction [33]. In addition, the concentration of C in the middle and upper parts of the coating is higher, where a large amount of TiC separates out and grows rapidly. The orientation of the grain growth is limited as grains restrict reciprocally while growing.

At the upper layer of the coating, part of the graphite combines with Ti to form TiC, while the other part of the graphite remained unreacted due to the reactions of Ni with the rest of Ti, which was unmelted and finally remained as these lath-like microstructures. Thus, it can be concluded that Ni has an excellent protection effect on the forming of a self-lubricating graphite phase.

### 3.3. Micro-Hardness and Wear Resistance

**Figure 6:**
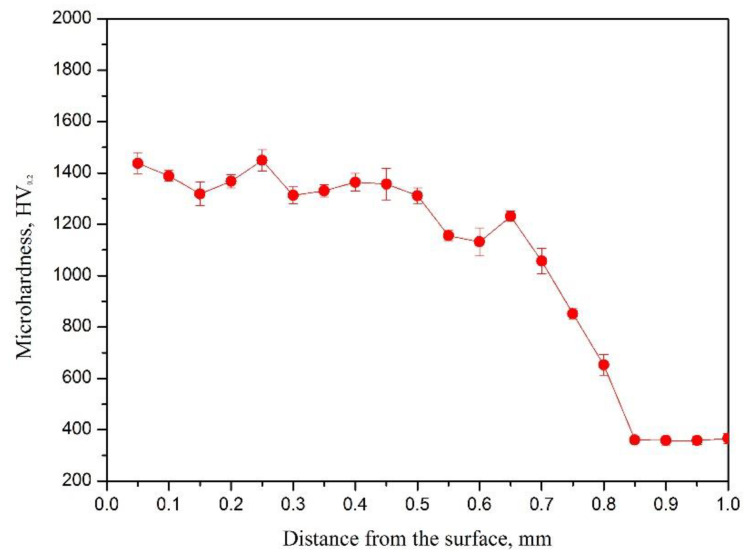
The hardness distribution of the coating.

The micro-hardness distribution along the cross-section of the coating is shown in Figure 6. The results have shown that the thickness of the coating is about 1.1 mm and the micro-hardness (1319.25 HV_0.2_) of the coating is about four times that of the Ti-6Al-4V substrate (334.52 HV_0.2_ [34]). The high micro-hardness can be attributed to the comprehensive impacts of dispersion strengthening (TiC) and solution strengthening (γ-Ni). It is noteworthy that there is an abrupt decrease at the bonding area between the coating and the substrate, which can be attributed to the fact that the composition was diluted by the melted substrate material. Compared with laser surface carburizing on Ti-6Al-4V [16], the hardness of the coating by laser alloying with G@Ni are higher due to γ-Ni solution strengthening.

**Figure 7:**
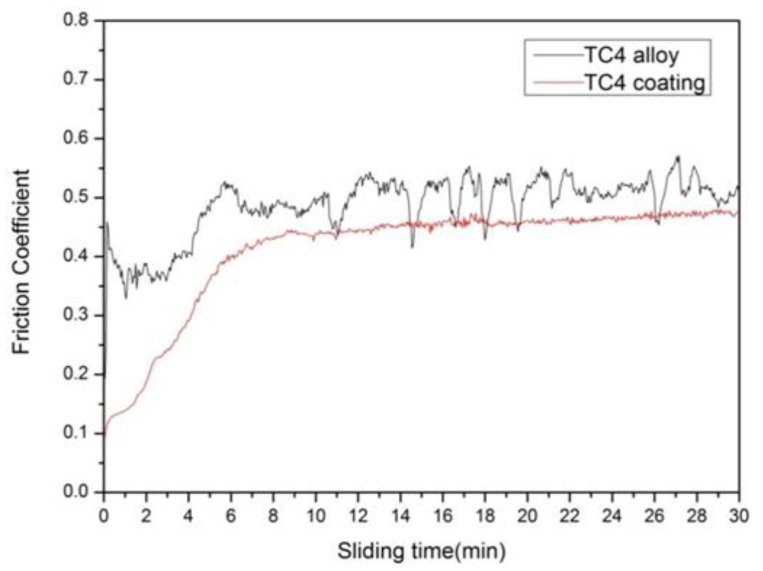
The friction coefficient curves of the coating.

**Figure 8:**
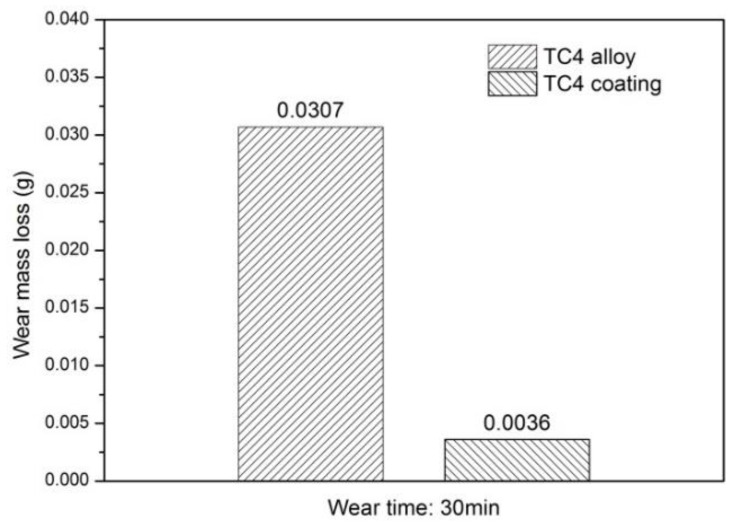
Wear mass loss of the substrate and the coating.

Figure 7 shows the friction coefficient of the Ti-6Al-4V substrate and the coating. The friction coefficient of the substrate is about 0.50, which is 0.05 higher than the coating whose friction coefficient is about 0.45. Since the coating contains lubricating-phase graphite, it is extruded under the application of external normal pressures, effectively lubricating the coating during the friction process. This results in a lower friction coefficient than the substrate. It can be seen from Figure 8 that the coating has a wear loss of 0.0036 g while the substrate has a wear loss of 0.0307 g, which is 8.53 times that of the coating, proving the excellent wear resistance of the coating. The high micro-hardness of the coating effectively resists the micro-crowding of the micro-convex body and the wear debris on the surface of the grinding ball. This then reduces the adhesion tendency between the friction pairs. In addition, the surface of the coating also presents a certain amount of graphite self-lubricating phase to form a lubricating transfer film on the surface of the friction pair.

**Figure 9:**
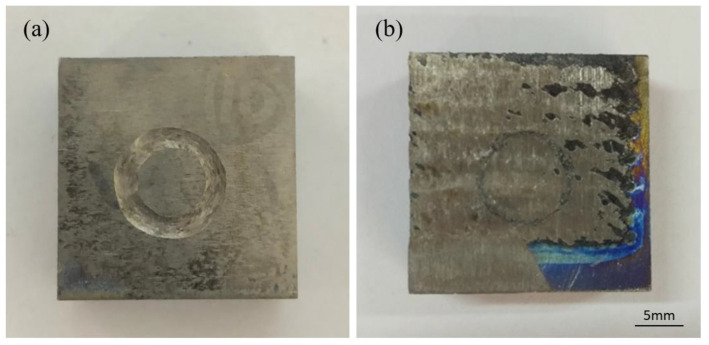
Surface morphologies of the substrate (**a**) and the coating (**b**) after the wear test.

**Figure 10:**
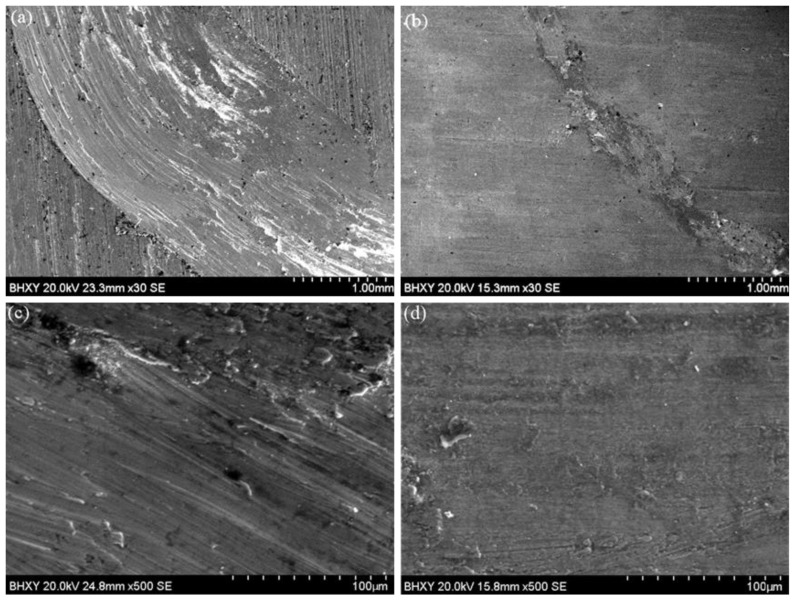
Wear surface morphologies of the substrate (**a**,**c**) and the coating (**b**,**d**).

Figure 9 shows the macroscopic morphologies of the Ti-6Al-4V substrate and the coating. The wear track of the coating has a much smaller width and depth than the substrate. There is only a shallow trace on the coating and a smaller amount of wear mass loss. Figure 9 shows the wear profile of the substrate and the coating after dry-sliding friction wear. The surface of the substrate presents deeper wear marks as well as obvious plastic deformations, grooves, and spalling. This is due to the fact that the hardness of the Si_3_N_4_ grinding ball is much higher than that of Ti-6Al-4V. In addition, when the grinding ball is pressed into the coating, the exposed TiC particles generate torque opposite to the motion of the grinding ball [35], effectively resisting the wear of the coatings. After continuous sliding friction, the micro-convex surface of the grinding ball surface is embedded into the low hardness substrate surface, leading to abrasive wears. In addition, the repeated deformation results in the spalling of the substrate. Figure 10 shows the wear surface morphologies of the substrate and the coating obtained by SEM, which proves that the surface of the coating is relatively smooth with the existence of a transfer film. Firstly, the TiC hard phase in the coating plays a major role in the wear resistance during the wear process, effectively suppressing the indentation of the micro-convex on the surface of the grinding ball. Secondly, the Ni-based solid solution in the coating has a higher toughness and is effective to prevent the generation and expansion of cracks. In addition, the graphite-lubricating phase forms a transfer film which converts the high-stress direct contact between the grinding balls and the coating into indirect contact to protect the surface of the sample.

## 4. Conclusions

A self-lubricating wear-resistant composite coating was prepared on the Ti-6Al-4V substrate with G@Ni powder by laser alloying. A good metallurgical bond was formed with the substrate. During the laser alloying process, a part of the graphite reacted with Ti to form TiC, while the rest remained unmelted due to the protection effect of Ni. Ni reacted with Ti to form NiTi and NiTi_2_, which improved the toughness of the coating. TiC, with petal-like or dendritic microstructures, was distributed in all layers of the coating and acted as the reinforcing phase. Graphite, at the same time, existed in lath-like structures only at the upper layer of the coating owing to its low density. Due to its outstanding self-lubricating effect, the graphite phase played an important role in friction reduction, while the TiC hard phase and the compact microstructures effectively increased the micro-hardness of the coating. The average hardness of the coating was about four times that of the substrate, and the result of the dry-sliding wear test shows that the wear resistance of the coating was enhanced to 8.53 times that of the substrate.

## Figures and Tables

**Table 1:** Properties of Ti-6Al-4V alloy and nickel-coated graphite.

Materials	Density (g/cm^3^)	Melting Point (°C)
TI-6Al-4V	4.5	1700
Ni	8.97	1455
G	2.62	3652

## Data Availability

The data presented in this study are available on request from the corresponding author.
